# The Moon and the Weather

**Published:** 1881-10

**Authors:** 


### THE MOON AND THE WEATHER.

M. de Parville has published in the *Journal des Débats* a paper on the temperature of the present year, which at Paris has risen to a height exceeding all previous authentic records. In the course of the article he raises the question as to whether the dryness of the present summer could have been foreseen, and answers it in the affirmative. Having then referred to the influence of solar action on the atmosphere, he says: "A very long series of observations has also shown that the moon, which passes every month from one hemisphere to the other, influences the direction of the great atmospheric currents. The changes in those currents, in consequence of the prevailing moisture or dryness, are intimately connected with the relative position for the time being of the sun and moon. The distance of the moon from the equator—that is, the inclination of the moon's path to the plane of. the equator —varies every year, passing from a maximum to a minimum limit; and the meteorological character of a series of years appears to be mainly dependent upon the change of inclination when those extreme limits have been touched. Observations prove that the rainy years, the cold winters, and hot summers, return periodically, and coincide with certain declinations of the moon. In our latitudes the rainy years occur when the moon's declination has touched its extreme limits of 28, 26 or 18 degrees respectively. They are separated from each other usually by periods of about three years, and then six years." M. de Parville then gives a list of rainy years running back to 1783, the most recent being 1879, 1876, 1872, 1866, 1859, 1856 and 1853, in each of which the moon's declination was either 28, 26 or 18, beginning with 26 degrees in 1879 and running back in the order named. The severe winters, he says, coincide as a rule within a year of the same declinations. The dry summers come naturally in the middle of the period which divides two wet years. The next wet year ought to coincide with the declination of 18 degrees, therefore with the year 1884, as the last was 1879 with the declination of 26 degrees. Between the two years comes the period of maximum dryness, and it may be expected therefore that the year 1882 will be another dry year.

### THINGS WE OUTGROW.

It is a happy experience for all of us to discover that we do not want half the things we hanker after.

It is both the curse and blessing of our American life that we are never quite content. We all expect to go somewhere before we die, and have a better time when we get there than we can have at home. The bane of our life is discontent. We say we will work so long and then we will enjoy ourselves. But we find it just as Thackeray has expressed it. "When I was a boy," he said, "I wanted some taffy. It was a shilling. I hadn't one. When I was a man I had a shilling, but I didn't want any taffy." —*Robert Collyer.*

The Mikado of Japan is to have a new palace at Yeddo, which will cost $5,600,000. Mike has evidently had some American contractors figuring on the job.—*Chicago Tribune.*

### MAKING TRADE FOR GRAVE DIGGERS.

From the objections that are made to one after another of the commonest articles of diet, it would appear that there is among us a number of base persons who are conspiring to make trade for grave diggers. There is not a single dish in common use but is soundly abused by some one ; bread is said to be worthless because it lacks the gluten of the wheat ; butter is said to be the cause of biliousnesss ; salt thickens the blood and makes the user cross ; milk softens the muscles and bones ; beefsteak contains prussic acid ; mutton is unfit to eat unless it has been kept so long that it is unfit to smell; pork means trichinæ; fish thins the blood; tomatoes produce cancer ; berries sour on the stomach, the coats of which are ruined by rhubarb stalks in pie or sauce ; green beans produce boils ; and the dreary list might be extended indefinitely.

Between all these calamities and death by starvation there is not much choice, unless one prefers to prolong a miserable existence by using some of the alleged foods that are put up in bottles with attractive lables. Yet somehow people go on eating all the dreadful things and living, and when any particular food product fails, and another is substituted, the health of the public does not seem to suffer by the change. If the people who buy family stores will pay less attention to talk about the healthfulness of various articles of food, and more attention to such methods of cooking as will make food most palatable and easy of digestion, there need be little fear of the natural quality of the rough material brought to the cook. It is dry, tough steak, the leathery slices of fried ham, the underdone vegetable and overdone meats, the greasy pies and heavy cake that play the mischief with the American digestion, health and temper.—*N. Y. Herald.*

### THE MONARCH MUMMIES OF EGYPT.

The obelisk which now stands in Central Park was hewn from the granite quarries at Assuan, in Upper Egypt, and erected at Heliopolis, the city of On of the Bible, by order of King Thothmes III., the fifth king of the XVIIIth Dynasty, who reigned B. C. 1600. The central columns of hieroglyphics of each of the four faces of the obelisk set forth the titles and honors of Thothmes III., whom Dr. Brugsch Pasha has designated as the Alexander the Great of Pharonic history. Two hundred and seventy years later (B. C. 1333), King Rameses II., surnamed Rameses the Great

(the Sesostris of the Greeks), ordered that his own titles and honors be inscribed upon the obelisk. The two outside columns of each of the four faces of the Central Park obelisk are composed of the hieroglyphics of Rameses the Great. Three thousand years later—that is to say, in the month of July, A. D. 1881—the mortal remains of these two monarchs were discovered in an almost perfect state of preservation in a cave cut in the solid rock near Thebes, where, together with the mummies of thirty other royal personages, they had been concealed by the Egyptian priests during those stormy days when Cambyses and his Persians overran Egypt and mutilated her sacred monuments.

Last June, Daoud Pasha, Governor of the Mudirieh of Keneh, noticed that the Bedaween brought in an unusually large quantity of relics, which they offered for sale at unusually low prices. This aroused suspicion, and by means of rewards and threats of corporal punishment the Pasha found out that what seemed to be an inexhaustible store of antiquities existed in a cave in the mountains near the Temple of Deir-el-Bahari, about four miles from the Nile to the east of Thebes. Daoud Pasha at once telegraphed the fact to Cairo, and the Khedive directed Herr Emil Brugsch, a younger brother of Dr. Brugsch Pasha (who, during the absence of M. Maspero, was in charge of the Boulak Museum), to proceed at once to Deir-el-Bahari and investigate the matter. The result is that Herr Brugsch has returned from his mission and safely landed in Cairo a steamboat full of the most precious relics of the Theban Dynasties. The following list of the principal objects of this "find" cannot fail to be of interest :

MUMMIES OF SOVEREIGNS, WITH THEIR WOODEN COFFIN CASES, JEWELS, CANOPIC JARS, ETC., INTACT.

| | Approx. date acc. to Brugsch. |
|---|---|
| 1. Aahmes I. (Amosis), 1st King of XVIIIth Dynasty | B. C. 1700 |
| 2. Amenhotop I. (Amenophis), 2d King of XVIIIth Dynasty | " 1666 |
| 3. Thothmes I., 3d King of XVIIIth Dynasty | " 1633 |
| 4. Thothmes II., 4th King of XVIIIth Dynasty | " 1600 |
| 5. Thothmes III., (The Great), 5th King of XVIIIth Dynasty | " 1600 |
| 6. Rameses I., 1st King of XIXth Dynasty | " 1400 |
| 7. Setee I., 2d King of XIXth Dynasty | " 1366 |
| 8. Rameses II., 3d King of XIXth Dynasty | " 1333 |
| 9. Pinotem I., 3d King of XXIst Dynasty | " 1033 |
| 10. Raskhenen (?) | |

Queens :

1. Ra-ma-ka (Hatasou ?)
2. Aahmes Nofert Ari.

Seventeen other princes and princesses, and among the latter Maut Nedjem, a daughter of Rameses the Great. Four papyri in a perfect state of preservation. One of these, that of Queen Ra-ma-ka, is most beautifully illustrated and illuminated in green and scarlet. It measures sixteen inches wide, and when unrolled its length will probably be found to measure 120 or 150 feet.

A magnificent tent made of pieces of leather of different colors and bearing the cartouche of King Pinotem I., with the royal vulture and stars in red, yellow and green.

Fifteen enormous wigs which were worn upon occasions of ceremony. These wigs are about two feet high, and are made of curled hair or wool falling in braids behind the back.

Three thousand seven hundred small porcelain funereal statuettes, each bearing cartouches and inscriptions.

Nearly two thousand other objects, such as drinking cups, baskets, vases, lamps, urns, chairs, dried fruits, boxes, etc., etc.

It is impossible to estimate the archæolog'c l value of these treasures until a careful inspection of them has been made and the papyri fully interpreted and compared with others. In the meantime the articles are carefully locked up in the Museum of Boulak, which is now necessarily closed to all visitors. M. Maspero is at present in Paris preparing for publication the texts of th pyramids of the Vth and VIth Dynasties, which were brought to light last winter. The text of the pyramid of Ounas (the last king of the Vth Dynasty) will be published at Paris in the next number of M. Maspero's *Receuil.* Fragments of this text, hitherto not supposed to have had any relation to each other, have been used in many later temples and tombs. This adds force to the growing conviction among Egyptologists that the earliest Egyptian civilization we know of is the highest, and that all that we know of it is its decadence. The oldest pyramid is the largest and best built; the oldest temple—that beside the Sphynx at Gizeh—shows masonry since unapproached; the oldest papyrus—though as yet hardly underst od—is the wisest, and the tombs and the temples of the Theban period are filled with extracts from ancient books not yet found complete. Three or four of these books furnish five-sixths of the texts of the Tombs of the Kings.

### ON GROWING FAT OR LEAN.

TO THE EDITOR OF THE SUN.—*Sir:* Having paid some attention to the subject of increasing and diminishing the amount of adipose tissue in the human frame, perhaps I may be permitted to say a word on the matter. It is quite true that certain kinds of diet are more fat forming than others, but it does not therefore follow that a person living on this kind of diet—say, for instance, buckwheat cakes and Barbadoes sugar—will become fat. In many cases which have come under my own observation, all attempts to increase the amount of fat by means of a special diet (carbonaceous) have ended in the person becoming bilious and suffering from a congested liver and constipation. On the other hand, I have known persons fatten on bread and water. The late Mr. Swaim, of Philadelphia, for many years of his life ate nothing but bread and molasses, yet he was a stout, healthy-looking man. Mr. Mechi, the eminent English agriculturist, according to his own statement, can increase his weight materially by merely taking an extra lump or two of sugar in his tea. Another gentleman became fat and ruddy cheeked by adding a pint of ale to his midday repast. But these are exceptional cases. As a general rule, if a person in good health, obtaining seven or eight hours' sleep per night, and not overworked, does not get fat on a mixed diet, he wont get fat at all, and neither buckwheat cakes and Barbadoes sugar, nor cod-liver oil, nor any other fat producer will fatten him. Sound sleep is one of the best fat producers that I know of, and then it has the advantage of increasing the general health and strength at the same time. Beware of altering your diet for the mere purpose of putting on more flesh. Results may follow that you dream not of.

It is easier to reduce flesh than to increase it, as a general rule. But there may be danger even here. Many of those obese gentlemen who followed the Banting system, and tried to live on lean meat, a very little bread, and a glass or two of sherry per day, found themselves with seriously disordered kidneys, the result of a highly nitrogenous diet. They might have obtained that leanness for which they sighed, and retained their health, had they reduced the total quantity of food consumed, and eaten less bread, potatoes and starchy food, and more turnips, cabbages, beets, cucumbers, tomatoes, melons, and other watery articles of diet, and taken more exercise and less sleep. As a rule, fat people are large eaters. They will tell you they are not. Don't believe them. Flesh and fat are not formed out of nothing. A stout youth of my acquaintance told me that he ate "a mere nothing," and only slept four hours per diem. I found that he really ate as much at one meal as I could eat at three, took little or no exercise, and slept nine or ten hours out of the twenty-four.

"Laugh and grow fat," is an old saying; but I have not found fat people, as a rule, mirthful, but on the contrary, rather grave. I mean the very fat, not the merely plump. I have, too, met with fat people who were exceedingly nervous, though it is generally supposed that nervous people must necessarily be lean. M. D.

### SANITARY DEPARTMENT OF "HALL'S JOURNAL OF HEALTH.

We are daily receiving requests from our readers to purchase for them "HEALTH FOODS," "FOOD MEDICINES," PURE BAKING POWDERS, and various MEDICINES that our experience has taught us the value of; also standard books; and last but not least, many families rely on us to procure for them PURE BOVINE VACCINE MATTER, which is the only vaccine matter that is always safe. These commissions already keep one person employed much of the time. We have therefore determined to make this a branch of our regular business, and announce ourselves ready to fill orders for GLUTEN of WHEAT, GLUTEN COFFEE, COOKED FOODS, as CRUSHED WHEAT, BARLEY, OATS, CORN and RYE, also the various preparations of MALT, and for books, medicines, vaccine matter and any other like articles that can be found in New York market.

E. H. GIBBS & CO.

THE mean depth of the sea is from four to five miles.

IT rains three times as often in Ireland as it does in Italy.

VENUS, Mars and Jupiter compare in size as a pea, a pin head and an orange.

THE mean height of the Englishman is five and a half inches above that of the Frenchman.

THE amount of common salt in the sea is estimated to be about five times the bulk of the Alps.

MR. ALCOTT is represented as once having defined "actuality" as "the thingness of here."—*Boston Post.*

A THERMOMETER, plunged into the snow to the depth of four inches, will mark nine degrees more heat than at the surface.

IN ordinary breathing a man's chest takes in at one breath about twenty cubic inches of air, the bulk of a full-sized orange.

IF the existing waters of the sea were increased but one-fourth it would drown the earth, with the exception of some mountain summits.

### LIFE INSURANCE WITHIN THE REACH OF ALL.

INSURING one's life, which most people deem a duty, has hitherto been a luxury too costly for people of moderate means. Life insurance companies, in order to accumulate their enormous surplus funds, in many cases amounting to millions of dollars, have fixed their rates of premiums at figures which, we repeat, are prohibitory.

The extravagance of what we may properly call this old system of insurance has driven great numbers of persons to the adoption of a newer and better system of life insurance, within the reach of those to whom it is of the greatest use, to wit, persons of moderate means. This system is strictly mutual, and therefore the safest of any ; since, after providing for the ordinary expenses of economical management, members are only taxed the small sums required from time to time to make up the losses by death ; thus members are not taxed three or four times as much as is necessary in order to establish an enormous surplus fund in which they have no interest beyond the amount of their insurance. We believe in the new plan of life insurance, and have shown our faith by taking policies of insurance in two associations. We will forward all necessary printed information to any reader of the JOURNAL who will take the trouble to write to us on the subject.

